# Mosaic UPD(7q)mat in a patient with silver Russell syndrome

**DOI:** 10.1186/s13039-017-0337-1

**Published:** 2017-10-17

**Authors:** Jiasun Su, Jin Wang, Xin Fan, Chunyun Fu, ShuJie Zhang, Yue Zhang, Zailong Qin, Hongdou Li, Jingsi Luo, Chuan Li, Tingting Jiang, Yiping Shen

**Affiliations:** 1 Department of Genetic and Metabolic Central Laboratory, Guangxi Maternal and Child Health Hospital, Guangxi Birth Defects Prevention and Control Institute, No 59, Xiangzhu Road, Nanning, China; 2 Department of Laboratory Medicine, Boston Children’s Hospital, Harvard Medical School, 300 Longwood Avenue, Boston, MA 02115 USA

**Keywords:** Silver-Russell syndrome, Maternal uniparental disomy, Mosaicism

## Abstract

**Background:**

Silver-Russell syndrome (SRS) is one of the imprinting disorders characterized by prenatal and postnatal growth restriction, relative macrocephaly, body asymmetry and characteristic facial features. ~ 10% of SRS cases are known to be associated with maternal uniparental disomy of chromosome 7 (UPD(7)mat). Mosaic maternal segmental UPD of 7q (UPD(7q)mat) is very rare, had only been described in one case before.

**Case presentation:**

We reported a second case of mosaic segmental UPD involving 7q. The patient presented with dysmorphic features including thin and short stature, triangular face, moderate protruding forehead, relative macrocephaly, fifth toe clinodactyly and irregular teeth, meeting the clinical diagnosed criteria of SRS. This case indicated that ~ 80% of mosaic UPD(7q)mat lead to the manifestation of main phenotypes of Silver-Russell syndrome.

**Conclusions:**

Our case support the notion that there are genes control postnatal growth on long arm of chromosome 7 and indicate that ~ 80% of UPD(7q)mat mosaicism level was contributed to the SRS phenotype.

## Background

The Silver-Russell syndrome (SRS; OMIM #180860) is one of the imprinting disorders characterized by prenatal and postnatal growth restriction, relative macrocephaly, body asymmetry and characteristic facial features. To date, more than 400 SRS cases have been reported since it was initially described by Silver et al. in 1953 [1] and Russell in 1954 [2]. The clinical and genetic heterogeneity of SRS make it difficult to define its clinical diagnostic criteria and genetic etiology. Several scoring systems for clinical diagnosis of SRS have been proposed [3–7]. Most recent proposal suggested that a patients can be considered to have likely SRS if at least four out of the following six criteria were met: (1) small for gestational age, birth length and/or weight ≤ −2SDS, (2) postnatal growth retardation (height ≤ −2SDS), (3) relative macrocephaly at birth, (4) body asymmetry, (5) feeding difficulties and/or body mass index (BMI) ≤ −2SDS in toddlers; (6) protruding forehead at the age of 1-3 years (Netchine-Harbison SRS Clinical Scoring System). About 10% of SRS case had maternal uniparental disomy of chromosome 7, so far about 60 SRS patients with matUPD(7) had been reported [8]. The imprinting genes on chromosome 7 are believed to be involved in the pathogenesis of the syndrome [9–13], the specific causal gene(s) are yet to be identified.

Five cases with segmental UPD(7q)mat in patients with SRS phenotype had been documented in literature since 2001. The only mosaic segmental UPD(7) that was reported by Reboul et al. in 2006 revealed a 7q21-qter mosaicism in a patient with SRS phenotype [14]. Identifying segmental UPD(7) in patients with SRS may help to narrow-down causal genes and regions.

Here, we reported a second case with mosaic segmental UPD(7q) mat, the patient presented with the main phenotypes of SRS [15]. We compared the clinical findings involving mosaic UPD(7q)mat documented in literature and the finding support the notion that imprinted genes on 7q contribute to the pathogenesis of SRS, even in a mosaic status.

## Case presentation

**Fig. 1:**
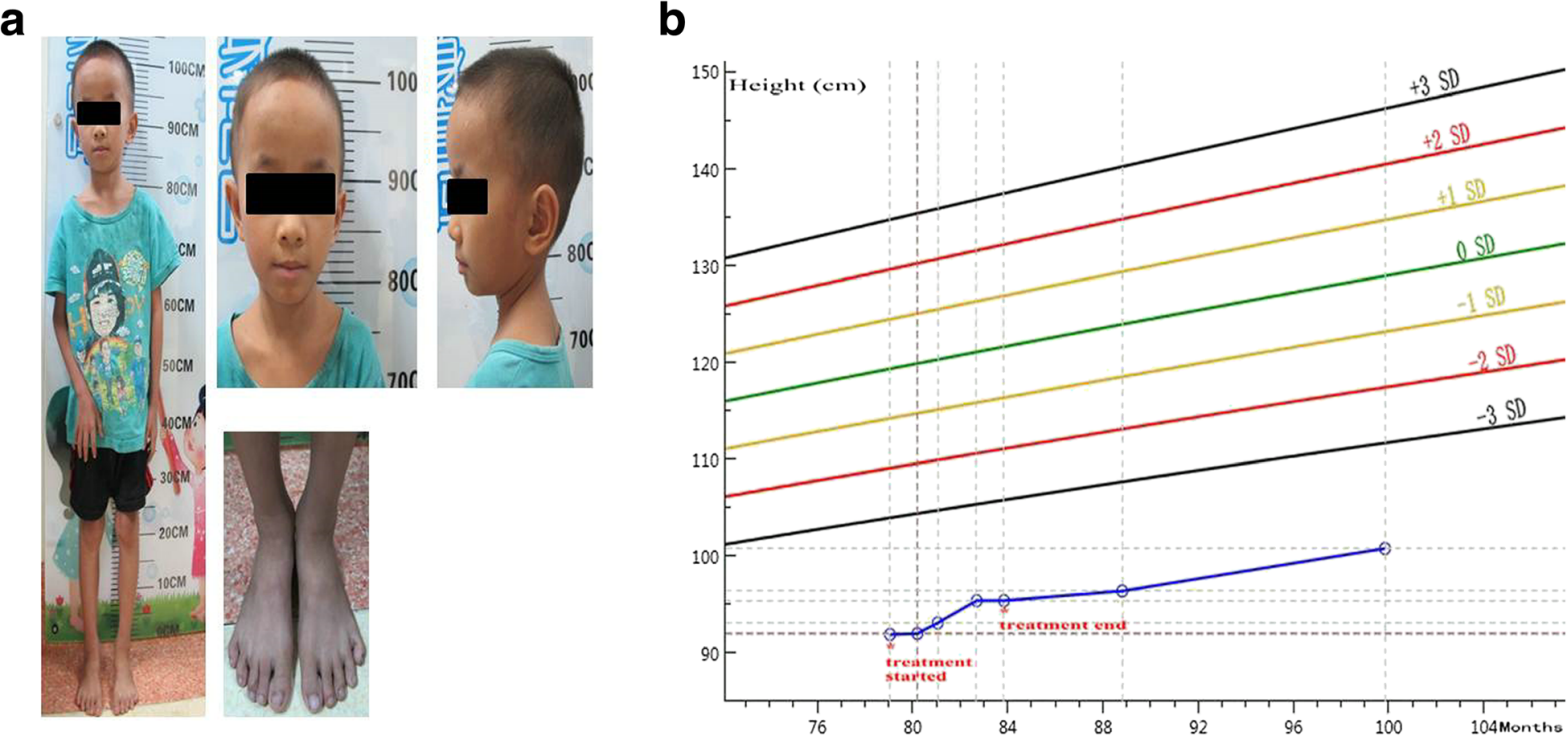
**a** Patient at age 8 years and 3 months. Dysmorphic features including thin and short stature, *triangular* face, moderate protruding forehead, relative macrocephaly, fifth toe clinodactyly and irregular teeth. **b** Growth curve of the patient: standard deviations (SD) were calculated based on the standardized growth charts for Chinese children and adolescents aged 0 to 18 [33], according to population-standard charts, the patient’s growth improved but remained ∼5.5 SD below the mean height for age

The patient was a six-year-old boy came to hospital due to severe developmental delay, short stature and mild dysmorphic features. He was a full term first child with two apparently healthy younger siblings. He was delivered via cesarean section without complication during pregnancy or delivery. His birth weight was 1.91 kg(<−3SD), indicating small for gestational age. His parents were non-consanguineous without family history of congenital anomalies. The paternal height was 162 cm, and the maternal height was 138 cm, the mother was not investigated for SRS and the height of the maternal familily members were not applied. The patient was reported to have failure to thrive since birth. During his initial hospital visit at the age of 6, his height was 91.9 cm (~ − 6 SD), weight 9.5 kg(~ − 6SD) and head circumference 49 cm (~ − 1SD, thus a relative macrocephaly). The bone age was delayed. Mild dysmorphic features were noticed including triangular face, moderate protruding forehead, relative macrocephaly, fifth toe clinodactyly and irregular teeth (Fig. 1a). Although the obvious body asymmetry was not noticed in this patient, this patient can be clinical diagnosed as SRS based on the Netchine–Harbison clinical scoring system (the first four features are present in this patient) [16]. In addition, he had normal electroencephalogram and electromyography, the growth hormone (GH) stimulating peak was 5.08 μg/L and diagnosed as partial growth hormone deficiency. He was subsequently treated with recombinant human growth hormone at the doses of 1 IU/(kg/d). The treatment lasted 4 months and stopped due to no significant growth response. Cytogenetic and molecular analysis were performed.

## Method

### Chromosomal microarray (CMA) and cytogenetic analyses

DNA sample was extracted from peripheral blood lymphocytes by using Lab-Aid DNA kit (Zeesan Biotech Co, Ltd., China). Genomic wide single nucleotide polymorphism (SNP) array analysis was performed by Illumina HumanSNPcyto-12 v2.1 BeadChip array. And the SNP data were collected and analyzed by Illumina Genome Studio and KaryoStudio software, cytogenetic analysis was performed by conventional standard GTG-banding at 400–550 band resolution.

### Microsatellite analysis

Eight highly informative microsatellite markers (short tandem repeat, STR) spanning the whole of chromosome 7 (D7S2552, D7S506, D7S510, D7S517, D7S672, D7S2410, D7S2504, D7S523) were selected from Genethon Genetic Maps (http://www.bli.uzh.ch/BLI/Projects/genetics/maps/gthon.html) for parent-of-origin analysis. The forward PCR primers designed for each STR maker were modified at 5′ terminal base with carboxyfluorescein (FAM). All fluorescent PCR products were analyzed on ABI 3130 genetic analyzer and GeneMapper (Applied Biosystems), the size and loci of each STR marker was assigned manually by identifying the peak on the electropherogram.

## Results

**Fig. 2:**
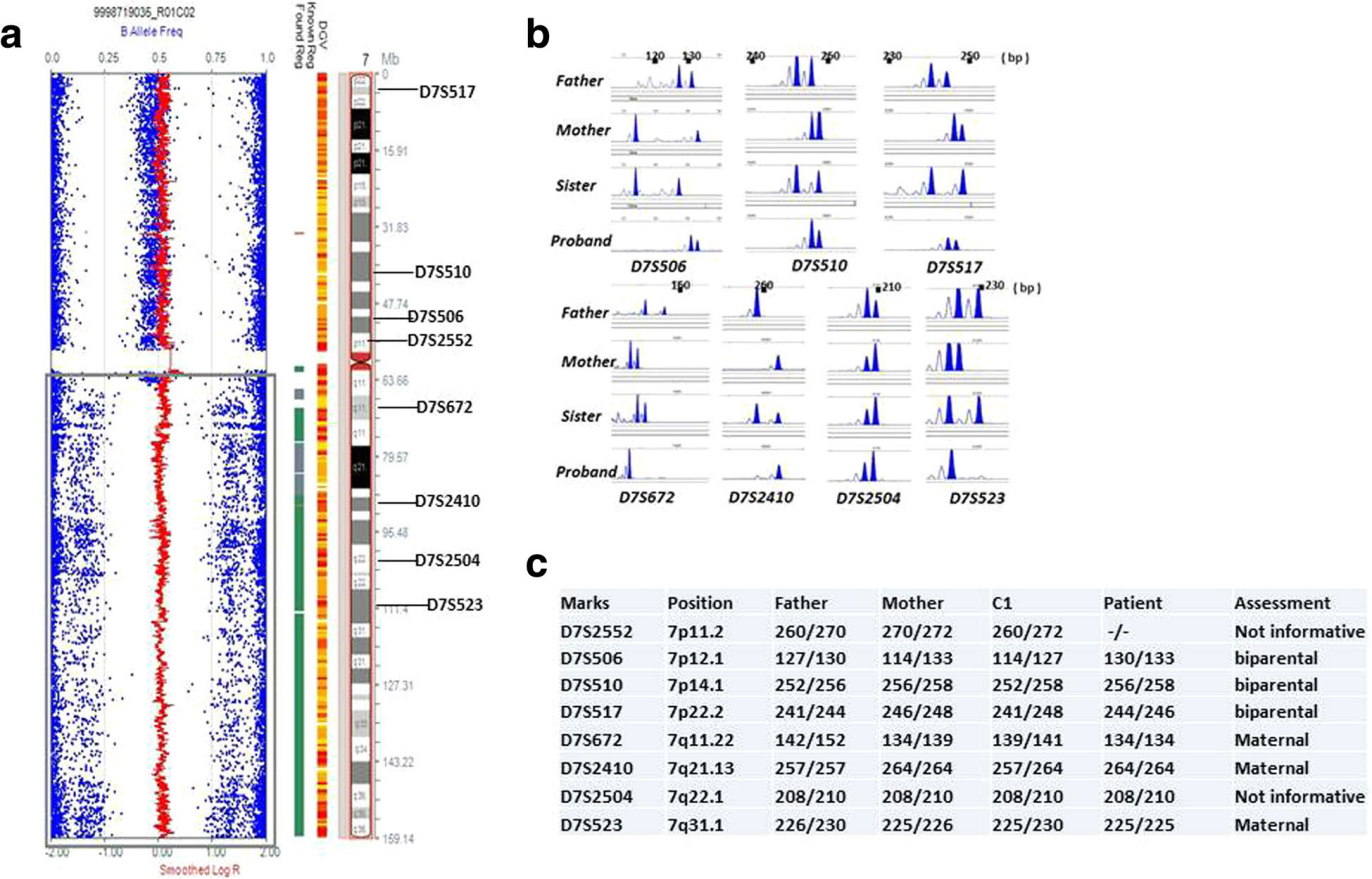
**a** The scatterplot representation of SNP microarray revealed the mosaic UPD region from 7q11 to 7qter. **b** Electropherograms of the D7S2552, D7S506, D7S510, D7S517, D7S672, D7S2410, D7S2503, D7S523 microsatellites in the proband (*bottom panel*), father (*top panel*) and mother (second panel). **c** The results of microsatellite analysis, the Arabic numbers denote the PCR product sizes in bp, D7S672, D7S2410, D7S523 loci revealed the UPD region detected by array was maternal origin

Uniparental disomy (UPD) of chromosome 7q was detected by CMA, spanned from the region of 7q11 to 7qter. The B allele frequency indicated that the UPD region was homozygous and mosaic (Fig. 2a), and the mosaicism level was estimated to be about 80% according to Conlin et al. [17]. G-band analysis presented normal karyotype. The STR makers analysis revealed that the UPD was maternal origin (Fig. 2b, c).

## Discussion

We reported a rare case of SRS due to mosaic segmental UPD(7q)mat. This is only the second such case ever reported [14] and the first case detected by SNP array. There are more than 60 UPD(7)mat cases documented in the literature since the first case of UPD(7)mat reported by Spence et al. [8, 18], we summarized the clinical features of typical SRS features and UPD(7)mat cases in Table 1. The body asymmetry phenotype was only ~ 39% prevalent among SRS cases. Relative macrocephaly (79.2%), SGA, BL and/or BW (61.6%), prominent forehead (61.6%), clinodactyly V digits (57.0%), feeding difficulties (46.5%), were the most common phenotype in the UPD(7)mat cases. Our patient presented with at least four common features, thus meet the clinical diagnosis of SRS (Netchine-Harbison SRS Clinical Scoring System, NH-CSS) [3].

**Table 1:** Phenotypic comparison of typical SRS cases and SRS due to UPD(7) mat

Clinical findings	SRS^*a*^ (*NH-CSS*)	UPD(7) mat cases^*b*^	mosaic UPD(7) mat cases^*c*^ *n* = 5	Our patient
Relative macrocephaly	+	61/77(79.2%)	3	+
SGA, BL and/or BW ≤ −2SD	+	53/86(61.6%)	5	+
Asymmetry	+	30/77(39.0%)	1	–
Feeding difficulties	+	40/86(46.5%)	2	–
Retarded bone age	+	24/77(31.2%)	1	+
Clinodactyly V digits	75%	49/86(57.0%)	3	+
Triangular face	94%	24/86(28.0%)	3	+
prominent forehead	+	53/86(61.6%)	3	+
Teeth anomalies	37%	14/77(18.2%)	1	+
Low set ears	49%	16/68(23.5%)	2	–
small chin	62%	10/86(11.6%)	1	+

Clinical findings: +, present; −, absent, *SGA* small for gestational age, *BL* birth length, *BW* birth weight

*NH-CSS* netchine-harbison SRS clinical scoring system

^a^data from Wakeling et al. [16], ^b^data from Kotzot, D. [34], Hannula et al. [35] and Fuke et al. [36], ^c^cases from Miozzo et al. [21], Bilimoria et al. [20], Flori et al. [22], Reboul et al. [14], and Tomoko et al. [23]

Mosaic UPD(7)mat was a rare condition. Monk et al. did not find any evidence of somatic mosaicism in three UPD(7)mat probands in lymphocyte and fibroblasts using both Southern blot and FISH analyses [19], subsequently, five mosaic UPD(7) mat cases have been reported (Table 2). Karl Y et al. found the first case of trisomy 7 (T7)/upd(7) mosaicism by amniocentesis, and the fetus showed likely-SRS symptoms with SGA, low-set ears, prominent forehead, triangular face, obstructed micrognathia, clinodactyly of fifth finger bilaterally [20]. Miozzo et al. used haplotyping and cytogenetic-FISH studies characterized a newborn presented SRS phenotype with complete maternal isodisomy 7 (i7) and trisomy 7 mosaicism [21]. Complete maternal heterodisomy 7 and trisomy 7 mosaicism was also reported by Elisabeth et al. [22]. Patient with full UPD(7) mat abnormal cell lineage and normal cell lineage without trisomy 7 also present SRS phenotype [23]. Reboul et al. reported a patient had UPD (7) without the mosaic trisomy 7 [14]. A summary of the clinical features of the five previously reported patients with mosaic UPD(7)mat and our present case is listed in Table 2. Collectively, these cases indicated that SRS phenotype could raise from mosaic UPD(7)mat status.

**Table 2:** Clinical features of mosaic UPD(7) mat cases

	Miozzo et al.	Bilimoria et al.	Elisabeth et al.	Reboul et al.	Tomoko et al.	Our patient
*Mosaicism UPD type*	Mixture of i7 andT7	Mixture of i7 and T7	Mixture of het 7 and T7	Mosaic segmental UPD7(q21-qter)	Mixture of i7 and N7	Mosaic UPD7(q11-qter)
*Evaluation analysis*	Microsatellite Metaphase FISH karyotyping	Microsatellite, karyotyping	Microsatellite, FISH	microsatellite analysis	microsatellite karyotype methylation	SNP array microsatellite
*tissuses*	Peripheral blood placental cotyledons	AF: ~ 27%^a^	AF: ~ 44% Intestine: 15% Skin: metaphases (5.5%) = nuclei (4%)	Peripheral blood	Peripheral blood: 92% Salivary:91%	Peripheral blood: ~ 80%
*Major Clinical findings*	IUGR, low birth weight, PNGR, relative macrocephaly, triangular face, prominent forehead, asymmetry	SGA, low-set ears, prominent forehead, small chin, triangular face, micrognathia, reversed epicanthal folds, clinodactyly of fifth finger bilaterally	Prominent large forehead, low osterior-rotated ears, small and retruded chin, bilateral clinodactyly of fifth fingers, VSD, PNGR, relative macrocephaly, feeding difficulties, triangular-shaped face, BAD	Growth failure, SGA, low birth weight, not show any craniofacial dysmorphic features.	Low birth weight/length, VSD, hydronephrosis, Feeding difficulty, speech delay, short stature, relative macrocephaly, abnormal teeth, 5th finger clinodactyly	Short stature triangular face, moderate protruding forehead, relative macrocephaly, fifth toe buckling and irregularly teeth.

*AF* amniotic fluid, *IUGR* intrauterine growth retardation, *PNGR* post-natal growth retardation, *SGA* small for gestational age, *VSD* ventricular septal defect, *BAD* bone age delayed

^a^the percentage in table stand for the mosaicism level

**Fig. 3:**
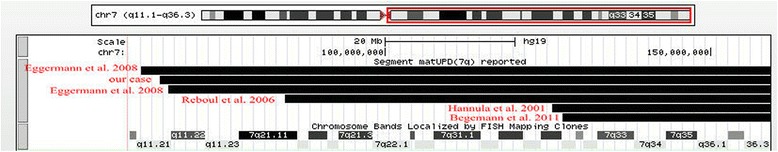
Schematic representation referred to segment mat UPD(7q) cases reported (UCSC genome browser custom track, hg19)

Five segmental UPD(7q) mat cases associated with SRS phenotypes had been documented in literature since 2001 [14, 24–26] (Table 3). The involved region would span the whole 7q and the smallest region was ~ 31 Mb to 7qter (Fig. 3). Clinical features of segmental UPD(7q)mat were compared in Table 3. The most common were relative macrocephaly and triangular face (5/5). Notably, psychomotor delay was present in the case reported by Eggermann et al. [25] and Begemann et al. [26], but not in the rest of cases. Additionaly, The relevance of 7p for the SRS phenotype currently known are maternal duplication of 7p12.1 (including gene *GRB10*) [27, 28], These findings are consistent with the notion that the imprinted gene(s) on chromosome 7q are the causes of SRS phenotype [9].

**Table 3:** The clinical features of segment UPD(7q) mat

	Hannula et al.	Reboul et al.	Eggermann et al.	Eggermann et al.	Begemann et al.	Total	Our patient
UPD region	D7S686 to qter	D7S2429 to qter	D7S663 to qter	D7S2429 to qter	chr7: 127,240,160-159,138,663	*N* = 6	chr7: 65,350,058-159,138,663 mos
Evaluation analysis	Microsatellite	Microsatellite	Microsatellite	Microsatellite	microsatellite		Array, microsatellite
gender	F	M	F	M	F	3F/2M	male
SGA	+	+	+	+	+	5/5	N
gestational age(weeks)	37.5	27	3 weeks before term	3 weeks before term	39		term
birth weight(g)	1510(−4.3SD)	600 (−3.5 SD)	2800 (−0.45SD)	2180 (−2.28 SD)	2410 (−2.74 SD)		1910
length(cm)	40 (−4.9SD)	N	46 (−1.16 SD)	45 (−1.97 SD)	44 (−3.7 SD)		N
head circumference	47 (0.2SD)	N	N	32 (−1.34 SD)	32 (− 2.77 SD)		N
age at evaluation(years)	1.35	33 months	5.3	1 4/12	3 2/12		6 7/12
weight(kg)	6.95	9.4 (−3.5 SD)	N	6.7 (−4.12 SD)	10.5 (BMI: 14.36)		9.5 (~ − 6SD)
height (cm)	71.5 (−2.9SD)	82 (−3.5 SD)	99.5 (−2.86SD)	73 (−3.60 SD)	85.5 (SD −3.09)		91.9 (~ − 6 SD)
feeding difficulties	–	–	+	–	+	2/5	–
relative macrocephaly	+	+	+	+	+	5/5	+
triangular face	+	+	+	+	+	5/5	+
protruding forehead	+	–	+	–	+	3/5	+
asymmetry	+	–	–	–	–	1/5	–
clinodactyly of the fifth fingers fingers/toes	+	–	–	+	+	3/5	fifth toe buckling
irregularly teeth	+	–	–	–	–	1/5	+
downturned mouth corners	+	–	–	+	–	2/5	–
ear anomalies	–	–	–	+	large prominent ears	2/5	–
development delay	–	–	–	+	+	2/5	–
other dysmorphic features	slender in appearance	–	–	single cafe’-au-lait spot	broad nasal bridge, ridge and tip, broad lips, retrognathia, epicanthal folds		slender in appearance

*F* female, *M* male, *N* not apply

The only previously reported a mosaic segment UPD(7q)mat from 7q21-qter mosaicism by Reboul et al. in a patient presented with SGA, relative macrocephaly, triangular face, severe growth retardation [14]. Our patient had only slightly larger size and presented with very similar phenotypes. The mosaicism level of Reboul’s case was not evaluated.

We detected and confirmed the mosaicism by both microsatellite and SNP array, the formulation to calculated the mosaic level proposed by Bliek et al. [29] by STR maker may be not accurate [30]. The pattern of B allele by SNP array offered an alternative and probable more accurate method for mosaic level assessment. Correlation between the level of mosaicism and SRS phenotype should be performed when more such cases are detected.

Complex and segmental UPD could resulting from either meiotic, mitotic, or meiotic and subsequent mitotic abnormal recombinations. Interestingly, apart from full upid(7)mat mosaicism which was likely result from mitotic non-disjunction and subsequent trisomy rescue, our case present the existence of normal cell line and UPD(7q11-qter) cell line, indicate the mosaicism may arise from somatic recombination [31, 32], further study would need to prove this hypothesis. Meanwhile, mosaicism could be tissue specific, but no more research for other tissue of our patient was performed because the father did not gave the consent.

## Conclusions

In summary, we described a second case with rare segmental maternal UPD(7q11-qter) mosaicism. This was the first report of UPD(7q11-qter) mosaicism detected by SNP array. Our case support the notion that there are genes control postnatal growth on long arm of chromosome 7 and indicate that ~ 80% of 7q11-qter mosaicism level was contributed to the SRS phenotype.

## Abbreviations

AF: Amniotic fluid
BL: Birth length
BW: Birth weight
CMA: Chromosome microarray analysis
IUGR: Intrauterine growth retardation
matUPD(7): Maternal uniparental disomy of chromosome 7
PNGR: Post-natal growth retardation
SD: Standard deviation
SGA: Small for gestational age
SNP: Single nucleotide polymorphisms
SRS: Silver-russell syndrome
UPD: Uniparental disomy
